# Glutamate Ionotropic Kainate Receptors as Therapeutic Targets in Enzalutamide-Resistant and Neuroendocrine Prostate Cancer

**DOI:** 10.3390/ijms27135945

**Published:** 2026-07-02

**Authors:** Huan Qu, Pengfei Xu, Joy C. Yang, Fan Wei, Junwei Zhao, Leyi Wang, Eva Corey, Nicholas Mitsiades, Kit Lam, Kenneth A. Iczkowski, Yuanpei Li, Allen C. Gao, Marc Dall’Era, Chengfei Liu

**Affiliations:** 1 Department of Urologic Surgery, School of Medicine, University of California, Davis, CA 95817, USA; hhqu@health.ucdavis.edu (H.Q.);; 2 Department of Biochemistry and Molecular Medicine, School of Medicine, University of California, Davis, CA 95817, USA; 3 Graduate Group in Integrative Pathobiology, University of California, Davis, CA 95817, USA; 4 Department of Urology, University of Washington, Seattle, WA 98195, USA; 5 Davis Comprehensive Cancer Center, University of California, 4645 2nd Ave, Research III Bldg, Suite 2300C, Sacramento, CA 95817, USA; 6 Department of Internal Medicine, School of Medicine, University of California, Davis, CA 95817, USA; 7 Department of Pathology and Laboratory Medicine, School of Medicine, University of California, Davis, CA 95817, USA

**Keywords:** prostate cancer, glutamate ionotropic kainate receptors, androgen receptor, enzalutamide resistance, neural lineage plasticity, NEPC

## Abstract

Treatment-induced neuroendocrine prostate cancer (t-NEPC) is the major form of resistance to androgen receptor signaling inhibitors (ARSI) in advanced prostate cancer, characterized by pronounced invasiveness and lineage plasticity. Through in-depth analysis of prostate cancer cohorts, we found that glutamate ionotropic receptor kainate (GRIK) family members, specifically GRIK2 and GRIK5, are highly expressed in neural lineage plastic prostate cancer cells, NEPC patient-derived xenografts (PDX), and NEPC patient samples. Their expression positively correlates with neuroendocrine markers and inversely correlates with androgen receptor (AR) activity. Additionally, functional analyses indicated that AR has a direct transcriptional inhibitory effect on GRIK2 and GRIK5, and the absence of AR signaling leads to the upregulation of GRIK2 and GRIK5. Further RNA sequencing analysis revealed that GRIK5 silencing reprograms the cellular transcriptome, resulting in significant downregulation of AR signaling and fatty acid metabolism, while simultaneously activating immune and inflammatory responses in enzalutamide-resistant prostate cancer cells. In both cell line and NEPC PDX organoid models, loss of GRIK5 impaired proliferation and clonogenic growth. Notably, GRIK5 also contributes to enzalutamide resistance. Pharmacological evaluation revealed that Pan-GRIK antagonists exhibit anti-tumor activity, although the required relatively high concentrations suggest that more potent therapeutic strategies should be developed. Collectively, this study establishes that GRIK family members play critical roles in enzalutamide resistance and NEPC progression, highlighting GRIK signaling as a potential therapeutic target for overcoming lineage plasticity in prostate cancer.

## 1. Introduction

Prostate cancer is a highly prevalent malignant tumor among men worldwide [1,2]. Although second-generation androgen receptor signaling inhibitors (ARSI), represented by enzalutamide, have significantly prolonged the survival of patients, most patients inevitably progress to the castration-resistant prostate cancer (CRPC) [3]. In recent years, the transition of advanced prostate cancer to treatment-induced neuroendocrine prostate cancer (t-NEPC) via lineage plasticity has been increasingly prevalent [4,5]. Tumor cells undergo a phenotypic transformation from a luminal cell state to a neuron-like neuroendocrine phenotype [6]. This reprogramming is characterized by the loss of AR signaling activity, acquisition of neural markers, and an extremely high potential for metastasis, for which effective targeted therapeutic options remain severely lacking in clinical treatment [5,7,8]. Previous research indicates that AR, as a key transcriptional regulator, directly binds to and represses the gene expression that drives lineage plasticity, such as BRN2, SOX2, and PLXND1 [9,10,11,12]. Upon ARSI-mediated treatment, which leads to impaired AR activity, these genes are activated and robustly upregulated, thereby initiating the neuroendocrine differentiation program.

The crosstalk between the nervous system and the tumor microenvironment is emerging as a frontier in oncology research [13,14]. Accumulating evidence has shown that malignant cells frequently appropriate neuronal signaling processes to support survival and proliferation [15,16]. Glutamate receptors, the main excitatory neurotransmitter receptors in the central nervous system, have demonstrated oncogenic potential across multiple non-neuronal systems. Among them, the glutamate ionotropic kainate receptor family (GRIK1-5) has been shown to be involved in the progression of various cancers. GRIK1 is highly enriched in recurrent glioblastoma and is associated with poor prognosis [17], whereas GRIK3 promotes epithelial-mesenchymal transition (EMT) in breast cancer via modulation of the SPDEF/CDH1 signaling pathway and functions as an oncogenic driver predictive of lymph node metastasis in gastric cancer [18]. In addition, GRIK5 has been found to stimulate colorectal cancer tumor growth and metastasis through the cAMP/PKA pathway [19]. These findings further support a broader role for glutamatergic signaling beyond the nervous system and suggest that components of this pathway may be reprogrammed during tumor evolution. Nevertheless, the functional contribution of ionotropic glutamate receptors to neural lineage plasticity remains largely unexplored.

In this study, analyses of public prostate cancer patient cohorts [20,21] revealed that GRIK family genes are enriched in neuroendocrine or neuroendocrine feature contexts, with GRIK2 and GRIK5 exhibiting marked overexpression. These findings suggest their potential involvement in the compensatory survival process following AR inhibition. Consistent with this notion, AR signaling intervention using DHT and enzalutamide demonstrated that GRIK expression is negatively regulated by AR, establishing functional relevance for this axis in lineage-plastic prostate cancer. Furthermore, transcriptomic profiling following GRIK5 silencing in enzalutamide-resistant cells identified GRIK5 as a regulator of gene programs involved in fatty acid metabolism, glycolysis, and immune-related pathways. Through integrated genetic and pharmacological studies, including dual knockdown of GRIK2 and/or GRIK5 in prostate cancer cell lines and organoid models, GRIK family proteins were identified as potential therapeutic targets for advanced NEPC. Collectively, these results define a previously unrecognized role for GRIK signaling in the transcriptional, metabolic, immune, and proliferative dependencies of NEPC and provide a rationale for targeting this pathway as a precision therapeutic strategy in advanced prostate cancer.

## 2. Results

### 2.1. GRIK Family Genes Are Significantly Upregulated in NEPC Patients and Neural Lineage Plastic Prostate Cancer Cells

**Figure 1:**
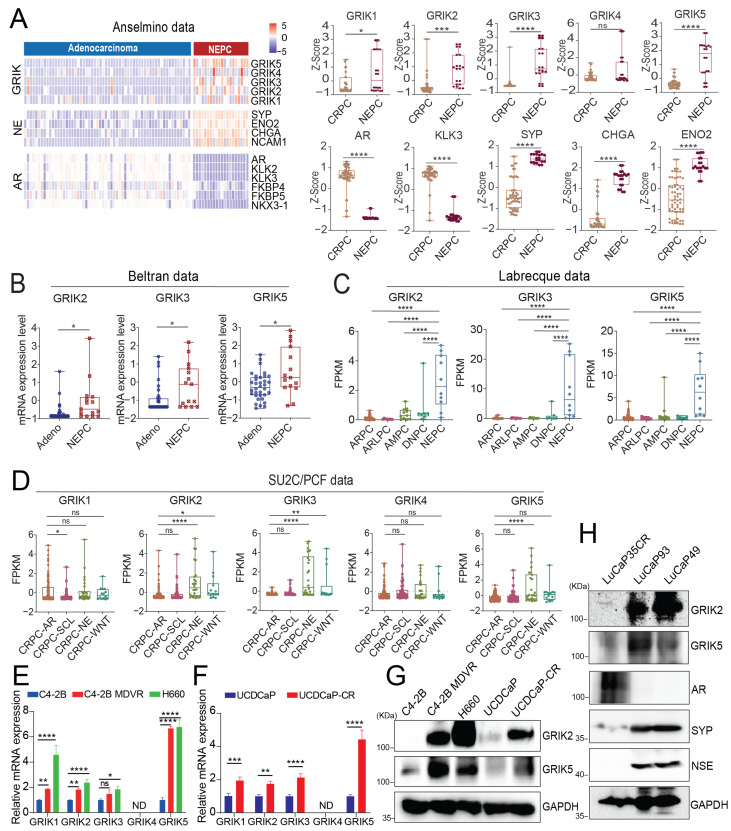
GRIK family genes are significantly upregulated in NEPC and associated with neural lineage plasticity. (**A**) Heatmap showing mRNA expression of GRIK family genes and neuroendocrine feature genes upregulated, and AR signaling key genes downregulated in NEPC from Anselmino datasets. (**B**,**C**) Quantitative analysis of GRIK family gene expression in different prostate cancer subtypes from the Beltran cohort and the Labrecque cohort. (**D**) Box-and-whisker plots depicting the mRNA expression of GRIK family genes across the different molecular subtypes in the SU2C/PCF cohort, including the androgen receptor-active (CRPC-AR), small cell-like (CRPC-SCL), neuroendocrine (CRPC-NE), and WNT-active (CRPC-WNT). Individual data points representing single patient samples. (**E**,**F**) mRNA expression of GRIK1, GRIK2, GRIK3, GRIK4, and GRIK5 in C4-2B, C4-2B MDVR, H660, UCDCaP, and UCDCaP-CR cells. (**G**) Western blot analysis of GRIK5 and GRIK2 protein expression in different prostate cancer cell lines. (**H**) Comparative protein expression of GRIK5, GRIK2, AR, NSE, and SYP in CRPC-PDX and NEPC-PDX tumors. Data are presented as mean ± SD. * *p* < 0.05, ** *p* < 0.01, *** *p* < 0.001, **** *p* < 0.0001, ns: not significant. ND: not detected.

To determine the expression patterns of GRIK family genes, we performed an analysis of multiple publicly available prostate cancer datasets. As shown in the Anselmino dataset [21], the mRNA expression of GRIK1, GRIK2, GRIK3, and GRIK5 is highly and specifically enriched in the NEPC subtype. Along with the established neuroendocrine feature markers such as SYP, ENO2, CHGA, and NCAM1, while being negatively associated with androgen receptor response markers such as AR and its target genes (Figure 1A). Consistent with these findings, the results of both the Beltran [20] and Labrecque [22] patient cohorts (Figure 1B,C) further verified the contrast in GRIKs expression between Adeno-CRPC and NEPC, with GRIK2, GRIK3, and GRIK5 showing significantly higher mRNA levels in the NEPC subtype compared to CRPC.

To further investigate the expression patterns of GRIK family genes in advanced prostate cancer, we analyzed the mRNA levels across the different subtypes in the SU2C/PCF CRPC cohort [23]. As shown in Figure 1D, GRIK2, GRIK3, and GRIK5 showed significant upregulation in the neuroendocrine subtype (CRPC-NE) compared to the androgen receptor-active (CRPC-AR) subtype; GRIK2 and GRIK3 were also increased in the WNT-active (CRPC-WNT) subtype, while there were no significant changes in the small cell-like (CRPC-SCL) subtype.

To confirm the clinical findings, we subsequently determined GRIK family gene expression in different prostate cancer cell lines and patient-derived xenografts (PDX) tumors. The results showed that enzalutamide-resistant or NEPC cell lines (C4-2B MDVR and H660) expressed high levels of GRIK1, GRIK2, and GRIK5, whereas GRIK3 expression was not significantly changed in the C4-2B MDVR cell line (Figure 1E). Similarly, UCDCaP-CR cells, a cell line showing neural lineage plasticity [24,25], exhibited significantly higher levels of GRIK1, GRIK2, GRIK3, and GRIK5 (Figure 1F). However, GRIK4 mRNA expression was not detectable in all these cell lines. Western blot analysis further revealed that both GRIK2 and GRIK5 protein levels were robustly elevated in C4-2B MDVR, H660, and UCDCaP-CR cells (Figure 1G). Additionally, we found that GRIK2 and GRIK5, together with classical neuroendocrine markers, were highly expressed in NEPC PDX tumors (LuCaP49 and LuCaP93) compared to CRPC PDX tumors (LuCaP35CR) (Figure 1H).

Taken together, these findings consistently suggest that GRIK family genes, particularly GRIK2 and GRIK5, are upregulated in NEPC. Importantly, their upregulation coincides with the loss of AR signaling and the acquisition of neural lineage plasticity features.

### 2.2. GRIK Family Gene Expression Is Positively Associated with Neuroendocrine Markers and Negatively Correlated with AR Signaling

**Figure 2:**
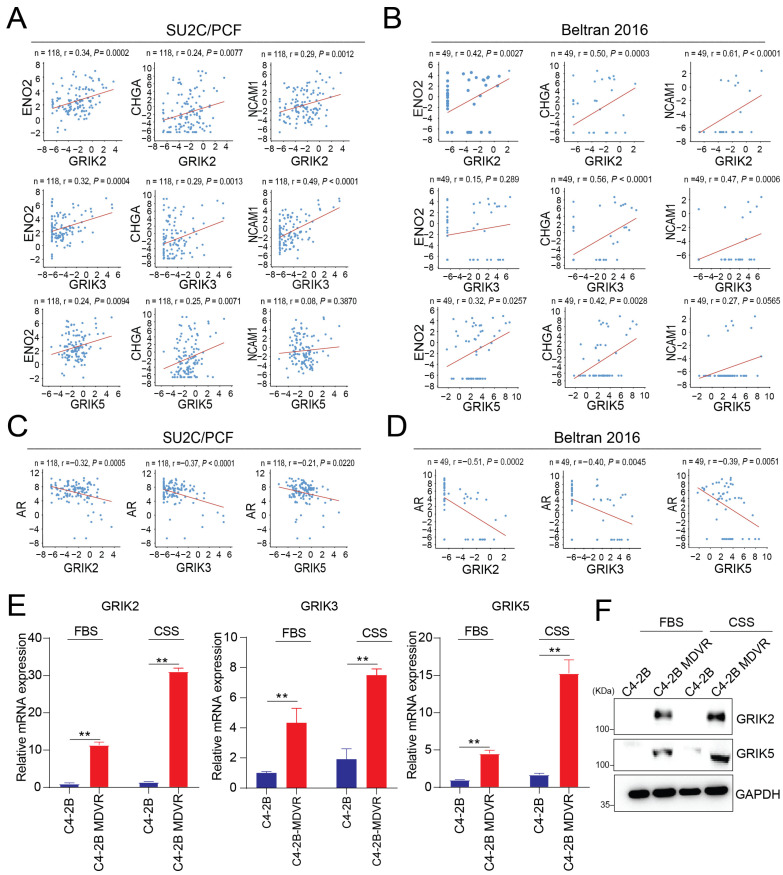
GRIK family gene expression is positively associated with neuroendocrine markers but negatively correlated with AR. (**A**,**B**) Co-expression of ENO2, CHGA, and NCAM1 mRNA (log2-transformed expression values) with GRIK family genes was assessed in the SU2C/PCF and Beltran 2016 [20] cohorts using Pearson correlation analysis. (**C**,**D**) Correlation analysis between GRIK family genes and AR mRNA expression (log2-transformed expression values) was performed in the SU2C/PCF and Beltran 2016 [20] cohorts using Pearson correlation analysis. (**E**,**F**) mRNA and protein expression of GRIK family genes in C4-2B and C4-2B MDVR cells cultured with regular FBS or CSS medium for 3 days. Data are presented as mean ± SD. ** *p* < 0.01.

To further determine the correlation between GRIK family genes and classical neuroendocrine features, the SU2C/PCF and Beltran datasets were analyzed. GRIK2 showed significant correlations with ENO2, CHGA, and NCAM1 in both datasets. GRIK3 was significantly correlated with ENO2, CHGA, and NCAM1 in the SU2C/PCF dataset, and with CHGA and NCAM1 in the Beltran dataset. GRIK5 was significantly correlated with ENO2 and CHGA in both datasets (Figure 2A,B). Conversely, GRIK2, GRIK3, and GRIK5 displayed significant negative correlations with AR expression in both datasets (Figure 2C,D).

To further explore the relationship between androgen signaling and GRIK family gene expression, we compared GRIK levels in C4-2B and enzalutamide-resistant C4-2B MDVR cells under different medium conditions. Since charcoal-stripped FBS (CSS) culture largely suppresses androgen activity, we found that relative mRNA levels of GRIK2, GRIK3, and GRIK5 were significantly higher in C4-2B MDVR cells compared to parental cells in both FBS and androgen-depleted CSS conditions (Figure 2E). Consistent with these findings, Western blotting confirmed that GRIK2 and GRIK5 protein levels were markedly elevated in C4-2B MDVR cells, particularly under CSS conditions, where AR signaling is minimized (Figure 2F). Overall, our results consistently indicate that GRIK family genes are negatively regulated by the AR signaling axis and are closely linked to the neuroendocrine phenotype in advanced prostate cancer.

### 2.3. AR Negatively Regulates GRIK Gene Expression in Prostate Cancer

**Figure 3:**
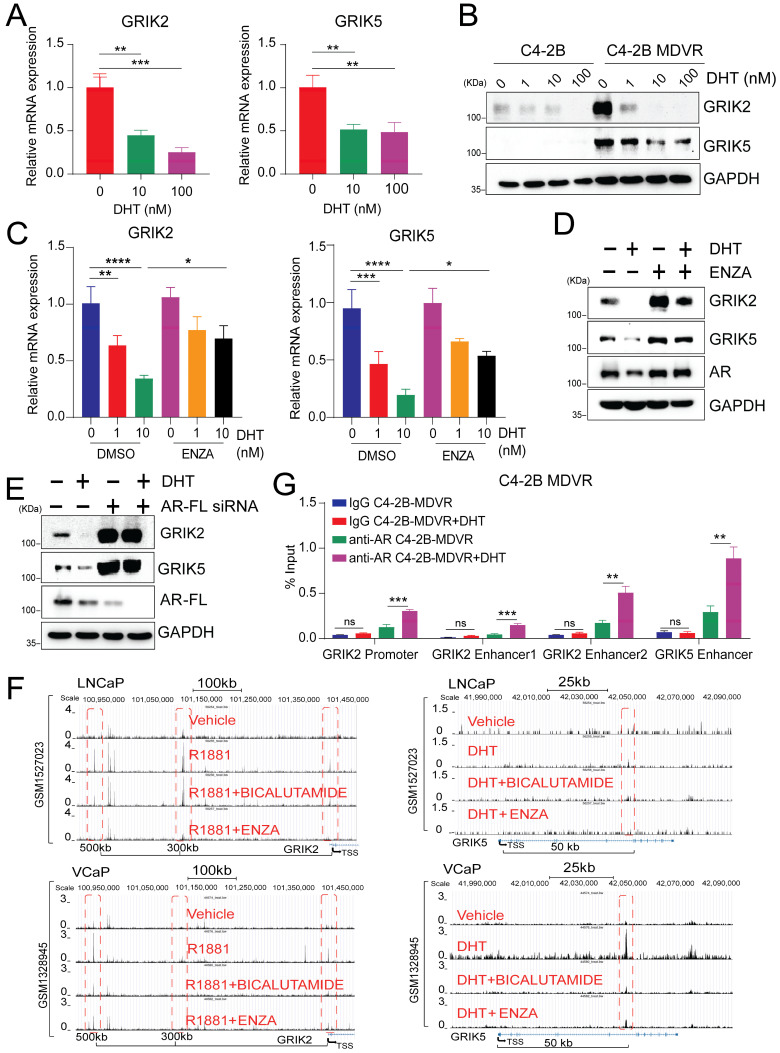
AR negatively regulates GRIK gene expression in prostate cancer. (**A**) C4-2B MDVR cells were cultured in CSS medium and treated with 10 nM and 100 nM DHT for 3 days. RNA was extracted and subjected to qRT-PCR analysis to determine the mRNA expression of GRIK2 and GRIK5. (**B**) C4-2B and C4-2B MDVR cells were cultured in CSS medium and treated with DHT at different doses (0–100 nM) for 3 days. (**C**,**D**) C4-2B MDVR cells were treated with DHT (0–10 nM) and/or enzalutamide (20 μM) for 3 days. Total RNA and protein were harvested for analysis. qRT-PCR and Western blotting were used to determine the mRNA and protein levels of GRIK2, GRIK5, and AR, respectively. (**E**) C4-2B MDVR cells were maintained in CSS medium, then subjected to siRNA-mediated AR knockdown for 3 days and treated with or without 10 nM DHT for 2 days. Western blotting was used to detect the protein expression of GRIK2, GRIK5, and AR-FL, respectively. (**F**) Visualization of ChIP-seq signals of AR enrichment across AR binding loci at the promoter or enhancer of GRIK2 and GRIK5 in LNCaP or VCaP cells treated with DHT/R1881 and/or the combination of bicalutamide/enzalutamide. (**G**) ChIP–qPCR analysis of the relative enrichment of AR at the indicated GRIK2 and GRIK5 loci in C4-2B MDVR cells treated with DMSO or DHT (10 nM) for 18 h. Data are presented as mean ± SD. * *p* < 0.05, ** *p* < 0.01, *** *p* < 0.001, **** *p* < 0.0001, ns: not significant.

To investigate whether AR signaling directly regulates GRIK expression, we treated C4-2B MDVR cells with increasing doses of DHT. As shown in Figure 3A, DHT treatment significantly suppressed the mRNA levels of GRIK2 and GRIK5 in a dose-dependent manner. This inhibitory effect was further confirmed at the protein level via Western blotting, where DHT treatment (0–100 nM) resulted in a robust decrease in GRIK2 and GRIK5 protein expression in both C4-2B and C4-2B MDVR cell lines (Figure 3B).

To further validate the requirement of AR activity in this regulation, we utilized the AR antagonist enzalutamide. In C4-2B MDVR cells, enzalutamide treatment partially rescued the DHT-mediated suppression of both GRIK2 and GRIK5 mRNA levels compared to the DMSO control group (Figure 3C). Correspondingly, Western blotting demonstrated that the reduction in GRIK2 and GRIK5 protein levels induced by DHT was attenuated upon the addition of enzalutamide (Figure 3D). To confirm that this regulation is specifically mediated by AR, we employed siRNA to knock down full-length AR (AR-FL). The knockdown of AR-FL significantly blocked the ability of DHT to suppress GRIK2 and GRIK5 protein levels (Figure 3E). These results were verified by Western blotting, showing that GRIK protein expression remained elevated despite DHT treatment when AR-FL was silenced. Finally, we analyzed available ChIP sequence datasets and found robust AR binding peaks enriched in the promoter and enhancer regions of both GRIK2 and GRIK5 following treatment with synthetic androgen R1881 or DHT. Conversely, the combination of AR antagonists (bicalutamide or enzalutamide) markedly reduced the AR-induced binding to the GRIK2 and GRIK5 gene loci (Figure 3F). Furthermore, we verified these findings in C4-2B MDVR cells by ChIP–qPCR. Indeed, a strong elevation of AR enrichment was observed at the GRIK2 promoter and two enhancer regions, as well as the GRIK5 intron enhancer region following DHT treatment (Figure 3G).

In summary, our data demonstrate that AR signaling negatively regulates the expression of GRIK2 and GRIK5 at both transcriptional and translational levels. These findings further suggest that the loss of AR signaling during NEPC progression may facilitate the aberrant activation of these genes.

### 2.4. GRIK5 Silencing Rewires Gene Expression Programs in Enzalutamide-Resistant Prostate Cancer

**Figure 4:**
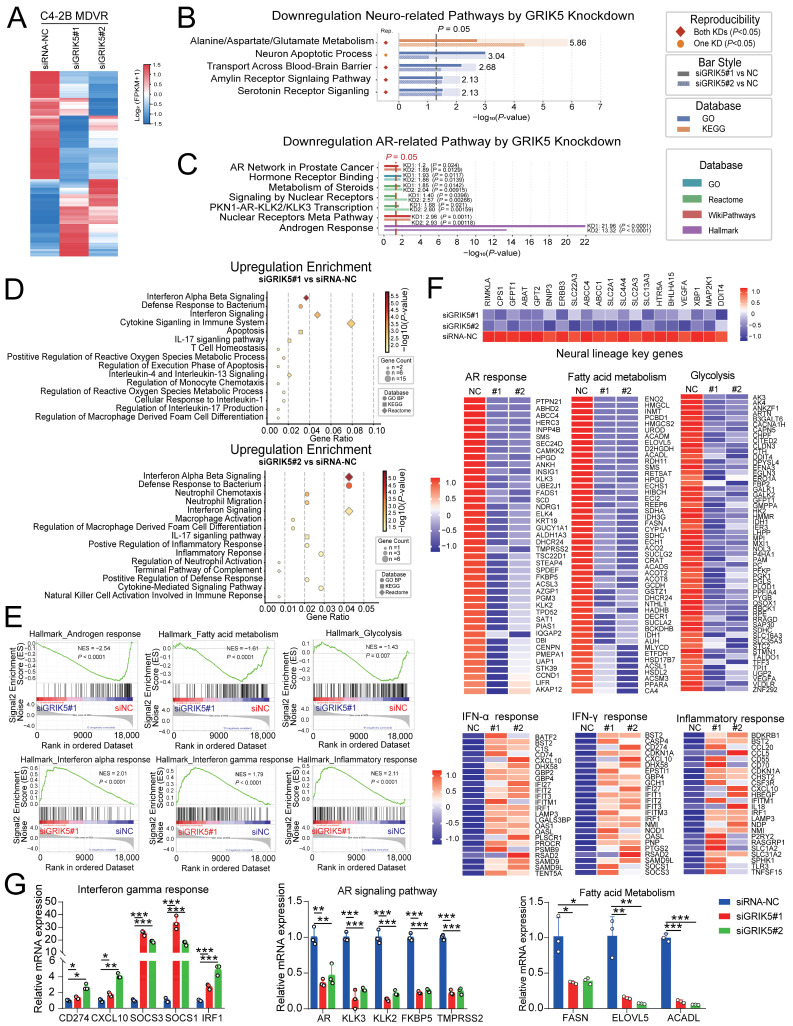
GRIK5 silencing rewires gene expression programs in enzalutamide-resistant prostate cancer. (**A**) Heatmap depicting the transcriptomic profiles of differentially expressed genes (DEGs) across the three experimental groups with Log_2_ fold change > 1.0 or < −1.0, *p* < 0.05. (**B**,**C**) Enrichment analysis demonstrates the downregulated neuro-related and AR signaling pathways in GRIK5 knockdown C4-2B MDVR cells using Biomni Lab. (**D**) Gene Ontology (GO), Kyoto Encyclopedia of Genes and Genomes (KEGG), and Reactome analysis showing upregulated pathways in GRIK5 knockdown C4-2B MDVR cells. (**E**) RNA-seq data from siGRIK5 and siRNA-NC-transfected C4-2B MDVR cells were analyzed using GSEA enrichment plots. The IFN-α response, IFN-γ response, and inflammatory response pathways showed significantly enriched immune-related signaling in the siGRIK5 group compared with the siRNA-NC group. The androgen receptor response, fatty acid metabolism, and the glycolysis showed significant downregulation after GRIK5 knockdown. (**F**) Heatmap of DEGs in GRIK5 knockdown C4-2B MDVR cells compared with the siRNA negative control (siRNA-NC) group. The rows represent individual genes, while columns denote normalized expression counts across samples. Red and blue colors demonstrate upregulated and downregulated expression levels, respectively. (**G**) qRT-PCR analysis of indicated pathway genes in C4-2B MDVR cells transfected with siRNA of GRIK5 or NC. ACTB was set up as a control. Data are presented as mean ± SD. * *p* < 0.05, ** *p* < 0.01, *** *p* < 0.001.

To define the downstream core transcriptional program upon GRIK5 knockdown, we conducted RNA-seq analysis in C4-2B MDVR cells with silenced GRIK5. The heatmap illustrated significant changes in the gene expression profile, showing the consistent patterns across two siRNAs (siGRIK5#1 and siGRIK5#2). A total of 190 genes were upregulated, and 265 genes were downregulated by siGRIK5#1 knockdown. A total of 141 genes were upregulated, and 223 genes were downregulated by siGRIK5#2 knockdown (Figure 4A). Subsequent analysis revealed that five pathways related to the neural lineage were highly enriched, including the downregulation of Alanine/Aspartate/Glutamate Metabolism, Neuro Apoptotic Process, Transport Across the Blood–Brain Barrier, Amylin Receptor Signaling Pathway, and Serotonin Receptor Signaling (Figure 4B). Consistently, multiple AR-related pathways, including the Androgen Response, PKN1-AR-KLK2/KLK3 Transcription, and AR Network in prostate cancer, were significantly inhibited (Figure 4C). Further enrichment analysis using GO, KEGG, and Reactome databases revealed significant enrichment of upregulated biological pathways related to Interferon Alpha/Beta signaling, Interferon signaling, as well as inflammatory signaling after GRIK5 knockdown in C4-2B MDVR cells (Figure 4D). GSEA analysis revealed that several hallmarks were significantly suppressed by GRIK5 knockdown, including fatty acid metabolism and glycolysis (Figure 4E). Heatmap visualization (Figure 4F) further highlighted the downregulation of neural lineage pathway genes and key metabolic enzymes, including FASN, ELOVL5, and ACADL, in C4-2B MDVR cells. Moreover, the Androgen Receptor Response pathway was significantly inhibited. The expression of AR itself and key target genes, such as KLK3, KLK2, FKBP5, and TMPRSS2, was prominently reduced after GRIK5 silencing. These findings indicate that GRIK5 is necessary for AR-driven transcriptional reprogramming and metabolic processes. Conversely, GSEA revealed strong upregulation of immune-related pathways (Interferon alpha response, Interferon gamma response, and Inflammatory response) upon GRIK5 knockdown. qRT-PCR validation further confirmed the upregulation of interferon-stimulated genes (ISGs) and inflammatory mediators (CD274, CXCL10, SOCS3, SOCS1, and IRF1), and the downregulation of AR signaling (AR, KLK3, KLK2, FKBP5, and TMPRSS2) and fatty acid metabolism (FASN, ELOVL5, and ACADL) in siGRIK5-transfected C4-2B MDVR cells (Figure 4G). Collectively, these data confirm that GRIK5 reprograms gene expression patterns and rewires signaling pathways in resistant prostate cancer.

### 2.5. GRIK Family Proteins Regulate Cell Proliferation and Modulate Enzalutamide Sensitivity in Prostate Cancer

**Figure 5:**
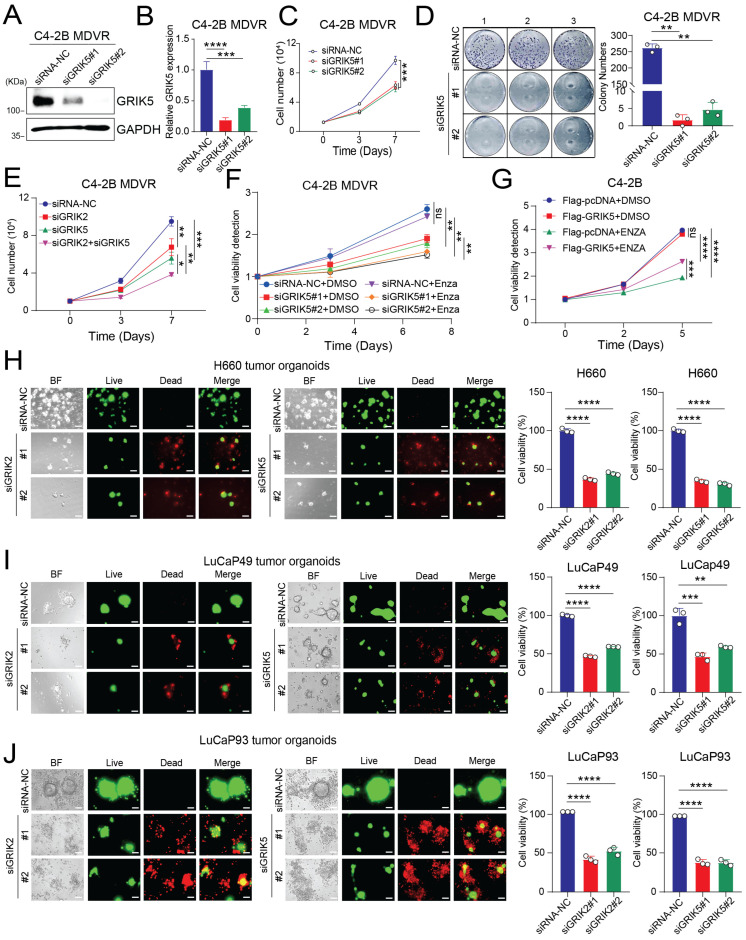
GRIK proteins control cell proliferation and enzalutamide sensitivity in prostate cancer. (**A**,**B**) C4-2B MDVR cells were transfected with either GRIK5-targeting siRNA or negative control, and GRIK5 knockdown efficiency was validated by Western blotting and qRT-PCR, respectively. (**C**) C4-2B MDVR cells (1 × 10^4^ cells/well) were seeded in 12-well plates and transfected with siGRIK5 or siRNA-NC. Cell counting was assessed on days 0, 3, and 7 post-transfection. (**D**) C4-2B MDVR cells were seeded in 6-well plates and transfected as indicated for the clonogenic formation assay, and colony numbers were subsequently quantified. (**E**) Cell proliferation curves of C4-2B MDVR cells transfected with siGRIK2, siGRIK5, or combined siRNA. Cell numbers were determined on days 0, 3, and 7. (**F**) Cell viability was determined in C4-2B MDVR cells treated with enzalutamide following GRIK5 knockdown. (**G**) Cell viability of C4-2B cells transfected with Flag-pcDNA or Flag-GRIK5 and treated with DMSO or 20 μM enzalutamide. Cell viability was measured on days 0, 2, and 5. (**H**–**J**) Organoids from H660/LuCaP49/LuCaP93 PDX tumors were seeded in 96-well plates (4 × 10^3^ cells/well) in 3D Matrigel and transfected with siGRIK5 and siGRIK2 or siRNA-NC for 9 days. Cell viability was determined by CellTiter-Glo assay, and the cells were visualized by LIVE/DEAD Cell Imaging Kit staining. Green color represents live cells, and red color represents dead cells. Scale bar = 100 μm. Data are presented as mean ± SD. * *p* < 0.05, ** *p* < 0.01, *** *p* < 0.001, **** *p* < 0.0001, ns: not significant. BF: bright field.

To evaluate the functional significance of GRIK signaling in advanced prostate cancer, we first performed siRNA-mediated knockdown of GRIK5 in the C4-2B MDVR cell line. Efficient suppression of GRIK5 was confirmed at both the protein (Figure 5A) and mRNA levels (Figure 5B). Functional assays revealed that silencing GRIK5 significantly inhibited the proliferation of C4-2B MDVR cells in a seven-day growth curve (Figure 5C). Colony formation assays further supported this phenotype, showing a dramatic reduction in both colony number and colony size following GRIK5 knockdown compared with that of the siRNA-NC control group (Figure 5D).

We next investigated whether GRIK family genes directly contribute to enzalutamide resistance. Simultaneous knockdown of GRIK2 and GRIK5 resulted in a more pronounced suppression of cell growth compared with individual knockdown (Figure 5E). Notably, although C4-2B MDVR cells are resistant to enzalutamide, silencing GRIK5 significantly sensitized these cells to enzalutamide treatment, resulting in a marked decrease in cell viability compared with the siRNA-NC + enzalutamide group (Figure 5F). Conversely, overexpressing GRIK5 (Flag-GRIK5) in parental C4-2B cells enhanced cell viability and conferred relative resistance to enzalutamide treatment (Figure 5G).

The biological importance of GRIK2 and GRIK5 was further validated using H660 tumor organoid and PDX tumor organoid models. In H660 organoids, siRNA knockdown of either GRIK2 or GRIK5 caused significant cell death, visualized by increased red fluorescence (dead) and reduced green fluorescence (live) (Figure 5H). Quantitative analysis showed that viability decreased by approximately 50% following GRIK silencing (Figure 5H, right panels). Similar results were observed in LuCaP49 and LuCaP93 tumor organoids, where targeting GRIK2 or GRIK5 significantly affected organoid maintenance and survival (Figure 5I,J).

In summary, these findings demonstrate that GRIK2 and GRIK5 are essential for the proliferation of NEPC-like cells and play a pivotal role in mediating resistance to enzalutamide, suggesting that they may serve as viable therapeutic targets in advanced prostate cancer.

### 2.6. Small-Molecule Inhibitors Targeting GRIK Protein Activity Suppress NEPC Proliferation

To explore the therapeutic potential of targeting GRIK activity, we evaluated the effects of two small-molecule inhibitors, DNQX and Perampanel, on various prostate cancer cell lines. Treatment with DNQX, a competitive antagonist of kainate/AMPA receptors, resulted in a dose-dependent reduction in cell survival across multiple models, including.

**Figure 6:**
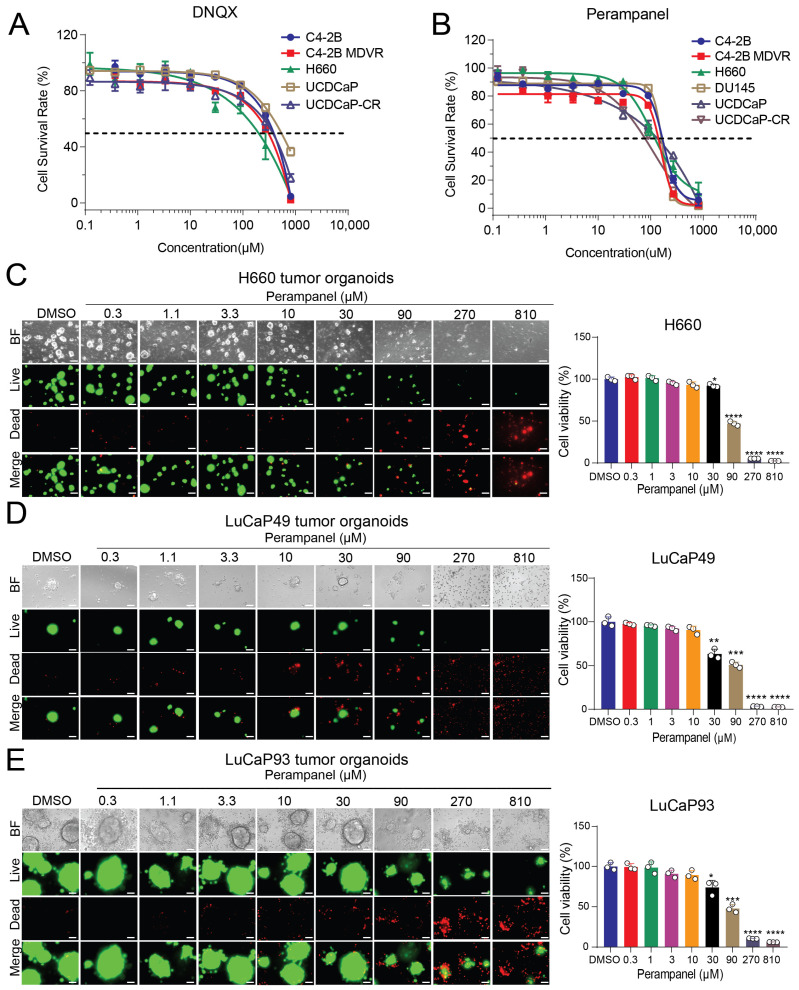
Small molecules that block GRIK protein activity potentially block NEPC proliferation. (**A**,**B**) C4-2B, C4-2B MDVR, H660, DU145, UCDCaP, and UCDCaP-CR cells were treated with different concentrations of DNQX or Perampanel compound for 3 days, and cell viability and IC_50_ values were determined by CellTiter-Glo assay. (**C**–**E**) Organoids from H660/LuCaP49/LuCaP93 PDX tumors were seeded in 96-well plates in 3D Matrigel and treated with Perampanel (0.3, 1.1, 3.3, 10, 30, 90, 270, 810 μM) for 9 days. Cell viability was determined by CellTiter-Glo assay, and the Live/Dead cells staining was visualized by LIVE/DEAD Cell Imaging Kit staining. Scale bar = 100 μm. Data are presented as mean ± SD. * *p* < 0.05, ** *p* < 0.01, *** *p* < 0.001, **** *p* < 0.0001.

C4-2B, C4-2B MDVR, H660, and UCDCaP/UCDCaP-CR cells, with IC_50_ values typically ranging between 100 μM and 1000 μM (Figure 6A). Notably, Perampanel, a more potent non-competitive antagonist, demonstrated superior efficacy, shifting the survival curves to the left with IC_50_ values observed between 100 μM and 300 μM across the tested cell lines (Figure 6B). The anti-tumor activity of Perampanel was further validated using H660 tumor organoids and PDX tumor organoids. In H660 tumor organoids, Perampanel treatment induced a significant, dose-dependent decrease in cell viability, visualized by the loss of green fluorescence (live) and an increase in red fluorescence (dead) starting at concentrations as low as 30 μM (Figure 6C, top panels). At higher concentrations (270–810 μM), organoid viability was almost completely abolished (Figure 6C, top bar graph). Consistent results were observed in LuCaP49 and LuCaP93 tumor organoids, where Perampanel effectively compromised organoid structure and survival in a dose-dependent manner, with a significant drop in viability occurring at 90 μM and above (Figure 6D,E). In summary, these findings demonstrate that pharmacological inhibition of GRIK protein activity with small molecules like Perampanel can effectively suppress the proliferation and viability of NEPC cells and organoids, suggesting that GRIKs are druggable targets for the treatment of advanced NEPC.

## 3. Discussion

In advanced prostate cancer, the development of treatment-induced neuroendocrine differentiation has increasingly been recognized as one of the major mechanisms underlying resistance to ARSI and subsequent lethal progression [26,27]. This lineage plasticity enables tumor cells to bypass AR signaling, promoting disease progression and therapeutic evasion [28]. Several studies have further found that elevated expression of neuroendocrine-related genes is strongly associated with a shortened clinical prognosis [4,29]. Our study identified that GRIK family genes, especially GRIK2 and GRIK5, are highly expressed in NEPC tissues or PDX models compared to prostate adenocarcinoma. This upregulation correlates strongly with neuroendocrine markers, while showing a significant negative correlation with androgen receptor activity. Physiologically, GRIK2 and GRIK5 are key subunits of the glutamate ionotropic kainate receptor family, where GRIK5 requires co-assembly with other kainate receptor family members. In the nervous system, kainate receptors regulate excitatory synaptic transmission and neural plasticity [30]. Extensive evidence indicates that these specialized receptors assemble into heteromeric complexes that are essential for governing synaptic homeostasis within the central nervous system [31,32]. In cancer biology, neural signaling programs are increasingly recognized as adaptive pathways that tumors engage in response to therapeutic stress [25]. Our findings show that GRIK2 and GRIK5 are specifically enriched in NEPC and lineage plastic models, including C4-2B MDVR, H660, UCDCaP-CR, and LuCaP NEPC xenograft tumors. Within this framework, enrichment of GRIK2 and GRIK5 in NEPC models is consistent with the view that the enrichment of neural signaling components in therapy-resistant prostate cancer suggests that tumor cells undergo substantial phenotypic plasticity, accompanied by the activation of neuronal developmental programs that may contribute to disease progression and therapeutic adaptation. The emerging literature indicates that kainate receptor subunits could be involved in oncogenic or regulatory roles in various malignancies [33,34,35]. Indeed, previous studies have demonstrated that GRIK5 is aberrantly activated in colon cancer, where it drives proliferation through the cAMP/PKA/CADM3 signaling axis [35]. Similarly, in cervical cancer, GRIK5 has been characterized as an important factor in metastatic progression [36]. The increase in GRIK expression in NEPC may be interpreted not only as a marker of neural lineage plasticity but also as a part of the transcriptional program that emerges when androgen receptor signaling is suppressed.

To elucidate the molecular mechanism of GRIK5 enrichment in enzalutamide-resistant prostate cancer cells, we explored its transcriptional regulation by AR signaling. Our in vitro data indicate that androgen signaling exerts direct and/or indirect transcriptional suppression of GRIK2 and GRIK5. When AR activity was inhibited by enzalutamide treatment, this suppressive effect was relieved, leading to the marked upregulation of GRIK2 and GRIK5. Mechanistically, the regulatory pattern is highly consistent with those of previously reported AR-repressed factors such as PLXND1, BRN2, and AKR1C3, all of which are known contributors to neuroendocrine differentiation or therapeutic resistance [9,24]. This suggests that GRIK signaling is one of the mechanisms by which prostate cancer cells achieve survival compensation when responding to treatment stress, by hijacking neural signaling molecules. Subsequently, prostate cancer cells overcome treatment-induced stress and maintain cell survival, thereby facilitating the transition to an NEPC phenotype. It is well-established that enzalutamide treatment induces neuroendocrine features in prostate cancer cells, reflecting a dynamic lineage plasticity process associated with therapeutic resistance [7,12,20,37]. Consistently, we observed that this phenotypic transition towards the neuroendocrine-like state is marked by significant upregulation of GRIK genes. Although the association between AR-targeted therapy and lineage plasticity is established, the functional link between enzalutamide-induced neuroendocrine transition and GRIK gene expression remains unreported. The functional significance of this mechanism is underscored by our knockdown experiments. Our studies demonstrated that knockdown of GRIK2 and GRIK5 significantly inhibited the proliferation of neuroendocrine-like cells and organoids and reduced clonogenic formation. Combined suppression of GRIK2 and GRIK5 produced a stronger anti-proliferative effect, and GRIK5 silencing increased enzalutamide sensitivity in C4-2B MDVR cells. These findings support a functional requirement for GRIK signaling in maintaining the NEPC status and AR-independent characteristics.

DNQX is a classical AMPA/kainate receptor antagonist (or pan-GRIK inhibitor) [38], while Perampanel is characterized as a selective AMPA receptor negative allosteric modulator [39]. Although the pan-GRIK antagonists exert inhibitory effects against NEPC models, effective suppression required relatively higher concentrations (>100 μM), suggesting that GRIK family genes function beyond their classical ion channel activity in tumor cells. Instead, they may serve as non-ionotropic scaffolding molecules that facilitate the activation of downstream kinase pathways through direct protein–protein interactions [40,41]. Notably, the biological role of GRIK5 appears to be environment-dependent across different tumor types. In GI-tract carcinomas, its oncogenic role appears to be driven by metabolic signaling and proliferative control [41]. In contrast, its function in NEPC is deeply integrated into adaptive lineage programming and cellular plasticity mechanisms.

The transcriptomic results of GRIK5 silencing suggest that this pathway also communicates with AR, metabolic, and immune-related states. Although classical AR signaling is suppressed in enzalutamide-resistant C4-2B MDVR cells, AR remains highly expressed in this cell line, suggesting alternative modes of AR pathway activation. The mechanism by which GRIK5 knockdown decreases AR transcription requires further investigation. Metabolic reprogramming represents another hallmark of NEPC biology and is considered essential for sustaining both aggressive proliferation and neuroendocrine lineage commitment [42,43]. Compared with adenocarcinoma cells, NEPC tumors often show enhanced glycolytic activity and altered lipid metabolism. Our transcriptomic profiling and GSEA analysis demonstrate that GRIK5 knockdown significantly attenuates multiple metabolic programs, specifically fatty acid metabolism and glycolysis-related pathways. These findings suggest that NEPC cells rely on GRIK5-dependent signaling to support the high bioenergetic demands of membrane biogenesis and rapid ATP turnover. Furthermore, this metabolic reprogramming is closely linked to the immune microenvironment. GRIK5 silencing acts as a potent molecular switch that activates pro-inflammatory signaling cascades to convert the immunologically ‘cold’ enzalutamide-resistant microenvironment into an ‘inflamed’ phenotype, including the IL-6/STAT3 and chemokine axes [44]. Our data suggest that GRIK5 influences tumor cell-intrinsic immune-related gene expression programs, and its inhibition could function as an agent to sensitize aggressive tumors to subsequent immunotherapy. This mechanism-based combination therapy warrants further clinical validation as a potential solution for patients who are unresponsive to conventional treatments.

## 4. Materials and Methods

### 4.1. Compounds

Perampanel was purchased from Cayman Chemical (Ann Arbor, MI, USA). Enzalutamide was purchased from Selleck Chemical (Houston, TX, USA). DNQX was purchased from MedChemExpress (Monmouth Junction, NJ, USA). Dihydrotestosterone was purchased from Sigma-Aldrich (St. Louis, MO, USA). All the chemicals were dissolved in dimethyl sulfoxide (DMSO).

### 4.2. Cell Lines and Patient-Derived Xenograft (PDX) Tumor Organoid Culture

C4-2B cells were kindly provided and authenticated by Dr. Leland Chung’s laboratory at Cedars-Sinai Medical Center (Los Angeles, CA, USA) and maintained in RPMI-1640 medium (Corning, Corning, NY, USA) supplemented with 10% FBS (Sigma-Aldrich, St. Louis, MO, USA) and 100 U/mL penicillin-streptomycin (Gibco, Grand Island, NY, USA). C4-2B MDVR (C4-2B enzalutamide-resistant cell lines) were routinely cultured in the complete medium supplemented with 20 μM enzalutamide to preserve the resistant phenotype [45]. C4-2B parental cells were passaged concurrently with the resistant cells as controls. H660 cells were obtained from the American Type Culture Collection (ATCC, CRL-5813) and cultured in RPMI-1640 medium containing 5% FBS, 10 nM hydrocortisone, 10 nM beta-estradiol, 5 μg/mL insulin, 10 μg/mL transferrin, 30 nM sodium selenite, and 1 × GlutaMAX (Gibco, #35050-061). UCDCaP and UCDCaP-CR cells were cultured in DMEM medium containing 10% FBS and 100 U/mL penicillin–streptomycin. The UCDCaP and UCDCaP-CR cell lines were established and characterized according to previously published protocols [24]. All cell lines were validated for authenticity through the short tandem repeat (STR) method and cultured at 37 °C in a 5% CO_2_-humidified atmosphere.

For organoid culture, H660, LuCaP49, and LuCaP93 PDX tumors were harvested and minced into small pieces (approximately 2–4 mm^3^). The tissues were subsequently digested with 5 mg/mL collagenase IV (Gibco, #17104019) in ADMEM/F12 medium (Gibco, #12634010) at 37 °C for 60 min until a single-cell or small-cluster suspension was achieved. The resulting cell suspension was filtered through a 40-μm cell strainer, followed by centrifugation and resuspension in ADMEM/F12 complete medium. The complete organoid medium was supplemented with 1% penicillin/streptomycin, 10 mM Hepes, 1 × GlutaMAX, and further enriched with a specific growth factor cocktail consisting of 1 × B27, 1.25 mM N-acetylcysteine, 50 ng/mL human recombinant EGF, 10 ng/mL FGF-10, 500 nM A-83-01, 10 μM SB202190, 10 mM nicotinamide, 10 nM dihydrotestosterone (DHT), 10 nM PGE2, 100 ng/mL Noggin, and 10% (*v*/*v*) R-spondin-1 conditioned medium. The isolated cells were mixed with Matrigel diluted 1:4 with complete ADMEM/F12 medium and seeded in 96-well tissue culture plates. Following 60 min incubation at 37 °C to ensure solidification, the organoid cultures were maintained under standard conditions overnight. After 24 h, the organoids were transfected with GRIK2 and GRIK5 siRNAs or treated with the small molecule Perampanel for 9 days. Cell viability was determined using the CellTiter-Glo Luminescent Assay (Promega, Madison, WI, USA, #G755B) and visualized using the LIVE/DEAD Cell Imaging Kit (Invitrogen, Carlsbad, CA, USA, #R37601) following the manufacturer’s instructions.

### 4.3. Plasmids and Cell Transfection

Small interfering RNAs (siRNAs) targeting GRIK5 sequences (Integrated DNA Technology IDT #1, Coralville, IA, USA (CUUCUUCCAGAAUUCACG) and #2 (GAUCAGACCAACAUCGAG)), GRIK2 (Integrated DNA Technology IDT #1 (UUUCUGACAGUGGAACGC) and #2 (UAGCUAAACAAACCAAGA)), or negative control (Invitrogen, #12935300) were synthesized from IDT Corporation. The siRNAs and negative control were transfected into C4-2B MDVR cells using Lipofectamine™ iMAX (Invitrogen, #13778150) following the manufacturer’s instructions. Knockdown efficacy was determined by qRT-PCR and Western blotting following transfection with GRIK5/GRIK2-targeted siRNAs. To establish GRIK5 overexpression models, C4-2B cells were transiently transfected with a Flag-GRIK5 plasmid (Sino Biological, Wayne, PA, USA, #HG20547-CF) using Lipofectamine™ 2000 and subsequently treated with enzalutamide for the indicated time.

### 4.4. Quantitative Real-Time PCR (qRT-PCR)

Total RNA was extracted from cells using the TRIzol reagent (Invitrogen) following the manufacturer’s protocol. RT-qPCR was performed on an BioRad CFX-96 real-time cycler, and target gene expression was normalized to ACTB, and relative gene expression was calculated using the 2^−ΔΔCt^ method [46]. The primers are shown in Table 1.

### 4.5. ChIP–qPCR and Data Analysis

Briefly, C4-2B and C4-2B MDVR cells were treated with DHT (10 nM) for 18 hours before being harvested. Chromatin fragments were sonicated using a Covaris E220 and precipitated for ChIP–qPCR. A ChIP assay was performed as described [47] and the antibody used was against AR (Santa Cruz Biotechnology, Dallas, TX, USA, #sc-7305) to verify AR binding to GRIK regulatory regions. Visualization of ChIP-seq signals at enriched genomic regions was achieved by using Cistrome DB (https://cistrome.org/db/#/, accessed on 10 April 2026). The primers are shown in Table 2.

### 4.6. Western Blotting Analysis

Whole cells were lysed in lysis buffer containing a proteinase inhibitor cocktail on ice. Protein concentration was determined using the Bradford Protein Assay Kit (BioRad, Hercules, CA, USA). Proteins were separated by SDS-PAGE and transferred to a nitrocellulose membrane (Millipore, Billerica, MA, USA). The membranes were blocked with 5% skimmed milk in P-buffered saline-Tween (PBST) for 1 h, then incubated at 4 °C overnight with the primary antibodies [anti-GRIK5 (1:1000, ABclonal, Woburn, MA, USA, #A15676), anti-GRIK2 (1:1000, ABclonal, Woburn, MA, USA, #A9722), anti-AR (1:1000 dilution, Santa Cruz Biotechnology, Dallas, TX, USA, #sc-7305), anti-SYP (1:1000, ThermoFisher, Waltham, MA, USA, #PA5-16417), anti-NSE (1:1000, Santa Cruz Biotechnology, Dallas, TX, USA, #sc-271384) and anti-GAPDH (1:1000, Cell Signaling Technology, Danvers, MA, USA, #2118)], respectively. The membranes were rinsed and incubated with the HRP-conjugated secondary antibodies (1:5000, Promega, Madison, WI, USA, #W4021) for 1 h at RT. The membranes were visualized with an Enhanced Chemiluminescent Detection kit (Millipore, Billerica, MA, USA) using the BioRad system (Hercules, CA, USA). The relative integrated density was measured and normalized against GAPDH protein expression.

### 4.7. Cell Growth Assay

C4-2B MDVR cells were seeded in the 12-well plates at a density of 1 ×  10^4^ cells/well and transfected with GRIK2, GRIK5, or a combination of GRIK2 and GRIK5 siRNA for 7 days. Total cell numbers were counted at days 0, 3, and 7. C4-2B cells were seeded in 96-well plates and transfected with Flag-GRIK5, then treated with enzalutamide for 0, 2, and 5 days. The viability of the cells was analyzed using the CellTiter-Glo assay (Promega).

### 4.8. Clonogenic Assay

C4-2B MDVR cells were seeded in 6-well plates at a density of 800 cells per well. After 14 days of siGRIK5 transfection, the colonies were fixed with 4% paraformaldehyde and stained with a solution of 0.5% crystal violet for 1 h. The number of colonies was then quantified for each group.

### 4.9. RNA-Seq Data Analysis

Total RNA was isolated from control and GRIK5-knockdown C4-2B MDVR cells using the RNeasy Mini Kit (Qiagen, Germantown, MD, USA) according to the manufacturer’s instructions, followed by DNase I treatment to remove genomic DNA contamination. RNA integrity and quality were assessed prior to library preparation. Polyadenylated mRNA was enriched from total RNA using oligo(dT)-attached magnetic beads and fragmented prior to cDNA synthesis. First-strand cDNA was synthesized using random hexamer primers, followed by second-strand cDNA synthesis. Sequencing libraries were generated using the Illumina TruSeq RNA Sample Preparation Kit and subjected to end repair, A-tailing, adapter ligation, PCR amplification, and purification. Library quality and concentration were evaluated using a Bioanalyzer, a Qubit fluorometer, and quantitative PCR.

The libraries were sequenced on an Illumina platform to generate 150-bp paired-end reads. Raw sequencing reads were filtered to remove adapter-contaminated reads, reads containing > 10% ambiguous nucleotides (N), and low-quality reads in which > 50% of bases had a Phred quality score < 5. Clean reads were aligned to the human reference genome (hg38) using HISAT2. Gene expression levels were quantified based on mapped reads and normalized as fragments per kilobase of transcript per million mapped reads (FPKM). Transcript assembly and abundance estimation were performed using StringTie. Differentially expressed genes were subsequently subjected to pathway enrichment analyses, including Gene Ontology (GO) and Kyoto Encyclopedia of Genes and Genomes (KEGG) analyses using Biomni Lab (https://biomni.phylo.bio/, accessed on 17 March 2026) or GSEA.

### 4.10. Gene Set Enrichment Analysis (GSEA)

Gene Set Enrichment Analysis (GSEA 4.1.0, Broad Institute, Cambridge, MA, USA) was performed with the Java application software (Java 14.0.2, http://software.broadinstitute.org/gsea/index.jsp, accessed on 18 March 2026) as described previously [47]. The GSEA software was used in pre-ranked mode with default parameters. Enrichment significance was defined by a normalized enrichment score (NES), a nominal *p* value < 0.05, and a false discovery rate (FDR) q value < 0.25. Pathways with a positive NES were considered significantly enriched. Furthermore, statistically enriched biological processes or pathways of DEGs were ranked and classified by the Biomni Lab for GO and KEGG pathways.

### 4.11. Datasets and Patients’ Cohort

Corresponding clinical annotations for both patient samples and patient-derived xenograft PDX models were accessed via the cBioPortal for Cancer Genomics platform (http://www.cbioportal.org/, accessed on 24 March 2026). Multiple independent cohorts were analyzed, including the Beltran 2016 cohort for castration-resistant and neuroendocrine prostate cancer (NEPC) [20], the SU2C/PCF Dream Team 2015 cohort for metastatic prostate cancer tumors [48] and the Anselmino cohort for prostate cancer PDX models [21].

### 4.12. Statistical Analysis

Statistical analysis was performed using GraphPad Prism 8.0 software. Raw data were summarized by means, standard deviations, and graphical summaries, and then transformed, if necessary, to achieve normality. Sample size was determined based on the power to detect significant differences (*p* < 0.05). No sample or data point from the analysis was excluded. Data are presented as mean ± SD from three independent experiments. Differences between individual groups were analyzed using a two-tailed Student’s T-test for single comparisons or one-way analysis of variance (ANOVA), followed by the Scheffé procedure for multiple group comparisons. The experiments and data processing were not blinded. Concordance between gene expression levels in clinical patient samples was determined using Pearson’s rank correlation. A *p* < 0.05 was considered statistically significant (* *p* < 0.05, ** *p* < 0.01, *** *p* < 0.001, **** *p* < 0.0001, ns: not significant).

## 5. Conclusions

Collectively, our data demonstrate that high expression of GRIK2 and GRIK5 in NEPC is driven by the loss of AR-mediated transcriptional repression. Beyond their roles in sustaining malignant proliferation, GRIK2 and GRIK5 function as critical mediators of lineage plasticity and therapy resistance. Importantly, GRIK signaling not only supports AR-independent growth but also coordinates metabolic and immune remodeling that enables tumor adaptation under therapeutic stress. Consequently, targeting the GRIK signaling axis emerges as a promising therapeutic strategy to circumvent ARSI resistance, suppress NEPC progression, and potentially reprogram the tumor microenvironment toward a more treatment-responsive state.

## Figures and Tables

**Table 1:** Real-time PCR primer sequences.

Genes	Primer Sequences (5′→3′)
GRIK1	F: TTCCCCCTTTTCCCTCCTCT
	R: CGTCTCCCTCATTCGTCCTT
GRIK2	F: CCTGAATCCTCTCTCCCCTGA
	R: GAGCACACAACTGACACCCA
GRIK3	F: AAACTCACCTGCCCCTCAAC
	R: CCATTTTCTGTGCCCTCTGC
GRIK4	F: CAAGACCGCCACCATCATCA
	R: TAGGCTGACACCATCCCAAG
GRIK5	F: GAGCGGGAGAAGGTCATCG
	R: CACACAGCAGGGGAGAAGG
CD274	F: TGGCATTTGCTGAACGCATTT
	R: AGGTCTTCCTCTCCATGCAC
CXCL10	F: TGAATCCAGAATCGAAGGCCA
	R: TGCATCGATTTTGCTCCCCT
SOCS3	F: CCAGTCTGGGACCAAGAACC
	R: TCGGAGGAGGGTTCAGTAGG
SOCS1	F: CCGACAATGCAGTCTCCACA
	R: AGGCCATCTTCACGCTAAGG
IRF1	F: ACCCTGGCTAGAGATGCAGA
	R: TCCTTGTTGATGTCCCAGCC
AR	F: CCTGGCTTCCGCAACTTACAC
	R: GGACTTGTGCATGCGGTACTCA
KLK3	F: GCCCTGCCCGAAAGG
	R: GATCCACTTCCGGTAATGCA
KLK2	F: CAACATCTGGAGGGGAAAGGG
	R: AGGCCAAGTGATGCCAGAAC
FKBP5	F: GGGAAGATAGTGTCCTGGTTAG
	R: GCAGTCTTGCAGCCTTATTC
TMPRSS2	F: CGTCGATTCTTGCCAGGGTG
	R: TAGCCGTCTGCCCTCATTTG
FASN	F: CTTCAAGGAGCAAGGCGTGA
	R: ACTGGTACAACGAGCGGATG
ELOVL5	F: TGTTCTGTGAGTTAGTAACAGGAGT
	R: CCAGAGGACACGGATAATCTTCA
ACADL	F: CGCCGCGCGATGTTCTC
	R: TCCACCAAGATGCTCTGCAA
ACTB	F: AGAACTGGCCCTTCTTGGAGG
	R: GTTTTTATGTTCCTCTATGGG

**Table 2:** ChIP–qPCR primer sequences.

Genes	Primer Sequences (5′→3′)
GRIK2 Promoter	F: GAAGACAGAACGTGTTGGGC
	R: GCGACTCCTTCAGTCCATGT
GRIK2 Enhancer1	F: CTCACATTGCAAACCTGTGGG
	R: TCACGATCTCACAAGTCCCAG
GRIK2 Enhancer2	F: GATTTCTTGACAAAGACACCAA
	R: TTCTGCTGAAAGTTTCTTTTGCT
GRIK5 Enhancer	F: CCAGAAACAAACGTCCATCTAG
	R: CATCGTACTGTGTGTTCTTTCTTG

## Data Availability

The RNA sequence data have been deposited in the GEO database under accession codes: GSE335562. The raw data generated for this study will be made available by the corresponding author upon request.

## Abbreviations

The following abbreviations are used in this manuscript:

ACADL	Acyl-CoA Dehydrogenase Long Chain
AMPA	Alpha-amino-3-hydroxy-5-methyl-4-isoxazolepropionic acid
AR	Androgen receptor
AR-FL	Full-length Androgen Receptor
ARSI	Androgen receptor signaling inhibitors
ATCC	American Type Culture Collection
ATP	Adenosine Triphosphate
CHGA	Chromogranin A
ChIP–qPCR	Chromatin Immunoprecipitation-quantitative Polymerase Chain Reaction
ChIP-seq	Chromatin Immunoprecipitation sequencing
CRPC	Castration-resistant prostate cancer
CRPC-AR	Androgen receptor-active Castration-Resistant Prostate Cancer
CRPC-NE	Neuroendocrine Castration-Resistant Prostate Cancer
CRPC-SCL	Small cell-like Castration-Resistant Prostate Cancer
CRPC-WNT	WNT-active Castration-Resistant Prostate Cancer
CSS	Charcoal-stripped FBS
DEGs	Differentially expressed genes
DHT	Dihydrotestosterone
DMSO	Dimethyl sulfoxide
DNQX	6 7-dinitroquinoxaline-2 3-dione
ELOVL5	ELOVL Fatty Acid Elongase 5
EMT	Epithelial-Mesenchymal Transition
ENO2	Enolase 2
FASN	Fatty Acid Synthase
FBS	Fetal Bovine Serum
FDR	False discovery rate
FKBP5	FKBP Prolyl Isomerase 5
FPKM	Fragments Per Kilobase of transcript per Million mapped reads
GO	Gene Ontology
GRIK	Glutamate ionotropic kainate receptor
GSEA	Gene Set Enrichment Analysis
ISGs	Interferon-Stimulated Genes
HRP	Horseradish Peroxidase
KEGG	Kyoto Encyclopedia of Genes and Genomes
KLK2	Kallikrein Related Peptidase 2
KLK3	Kallikrein Related Peptidase 3
MDVR	Enzalutamide-resistant cell lines
NC	Negative Control
NCAM1	Neural Cell Adhesion Molecule 1
NE	Neuroendocrine
NEPC	Neuroendocrine Prostate Cancer
NES	Normalized Enrichment Score
PDX	Patient-derived xenografts
PBS	Phosphate-Buffered Saline
qRT-PCR	Quantitative Polymerase Chain Reaction
RNA-seq	RNA sequencing
siRNA	Small interfering RNA
SYP	Synaptophysin
SDS PAGE	Sodium Dodecyl Sulfate Polyacrylamide Gel Electrophoresis
t-NEPC	Treatment-induced neuroendocrine prostate cancer
TMPRSS2	Transmembrane Serine Protease 2
